# Supplementary material for: MORC proteins regulate transcription factor binding by mediating chromatin compaction in active chromatin regions
Source: Genome Biol. 2023 Apr 26;24:96. doi: 10.1186/s13059-023-02939-4 (PMC10131428; doi:10.1186/s13059-023-02939-4)
Supplement: Supplementary file 1 — Additional file 1: Figure S1. Chromatin states of MORC7. Figure S2. Small RNA data over MORC7 peaks. Figure S3. Examples showing MORC7 enrichment over the promoter regions of the TOPLESS genes. Figure S4. MORC7 associates with some TFs. Figure S5. Metaplot and heatmap showing chromatin accessibility changes. Figure S6. Volcano plot showing TF changes for ZF off-target sites. Figure S7. Expression levels of genes in the primary shoot apical meristem specification pathway, with and without heat treatment, in Col-0 and morchex mutants. Figure S8. Motif enrichment of TPL and LUG peaks identified in Col-0 or morchex mutant. Figure S9. Metaplot and heatmap showing ChIP-seq signals over peaks detected in morchex mutant. [file 13059_2023_2939_MOESM1_ESM.pdf]

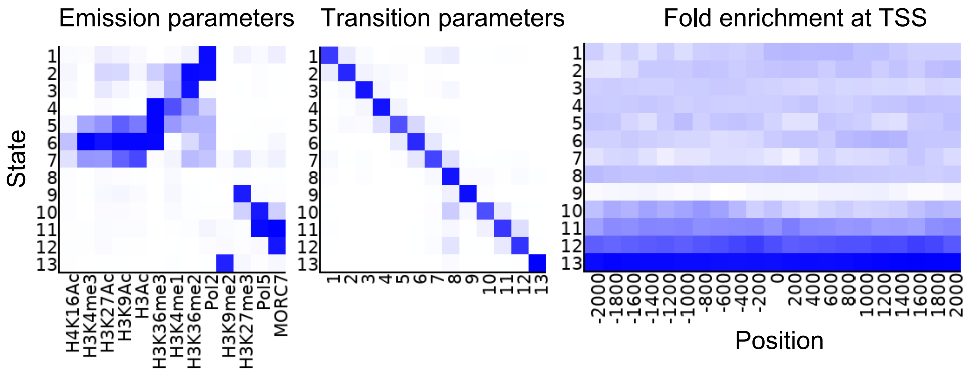

Fig. S1 Chromatin states of MORC7. ChIP-seq analysis was performed for H4K16ac, H3K4me3, H3K27ac, H3K9ac, H3Ac, H3K36me3, H3K4me1, H3K36me2, H3K9me2, H3K27me3, Pol II, Pol V, and MORC7.

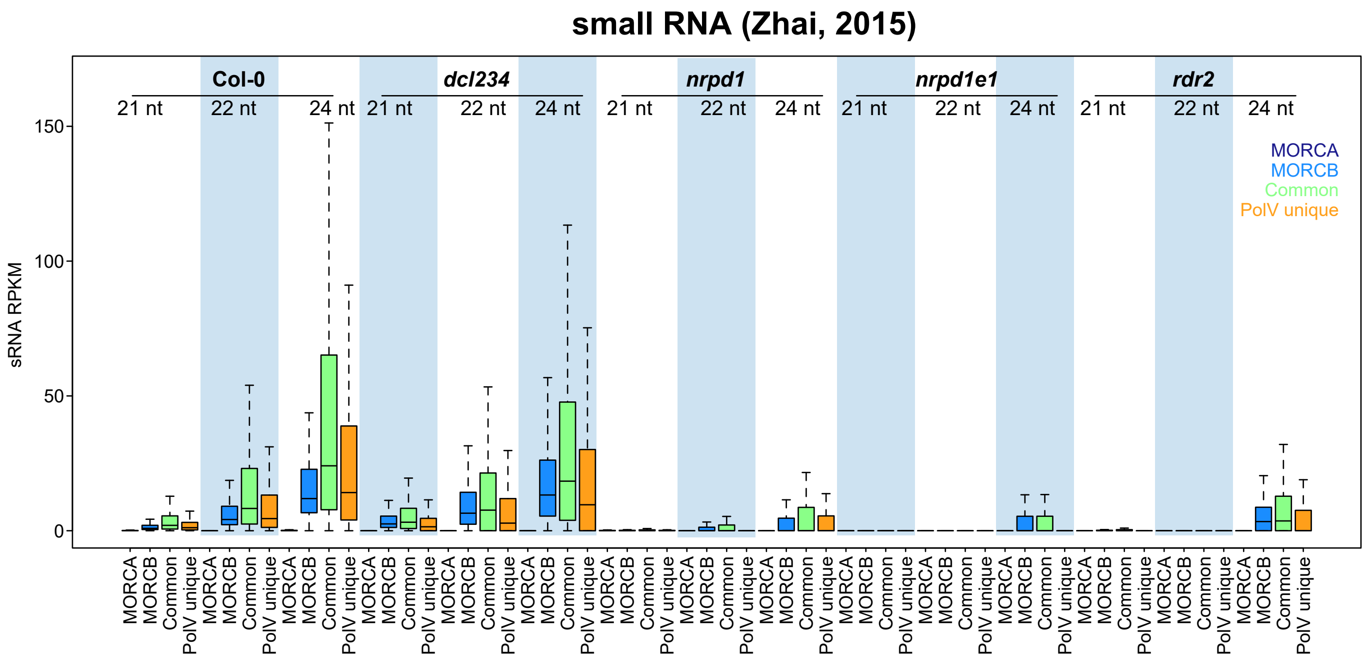

Fig. S2 Small RNA data over MORC7 peaks. Boxplot showing 21-nt, 22-nt, and 24-nt siRNA in Col-0, *dcl234*, *nrpd1*, *nrpd1 nrpe1*, and *rdr2* mutants [31] over MORC7A-unique, MORC7B-unique, MORC7-Pol V Common, and Pol V-unique regions.

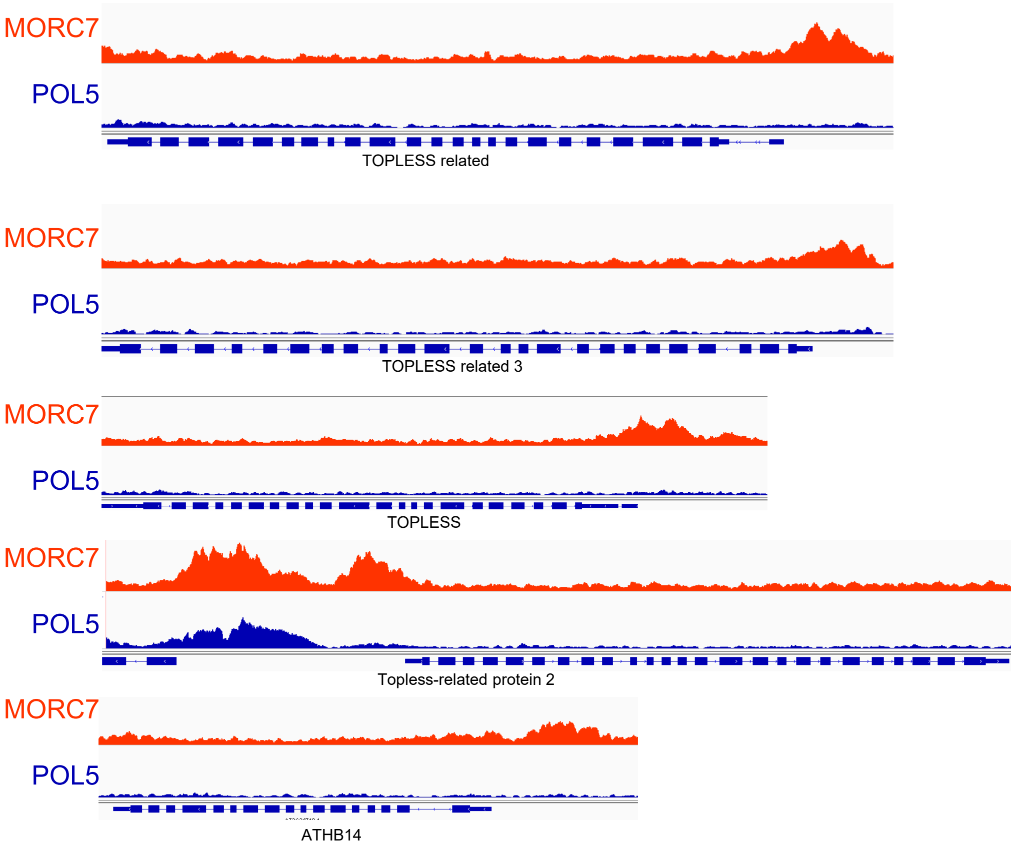

Fig. S3 Examples showing MORC7 enrichment over the promoter regions of the TOPLESS genes.

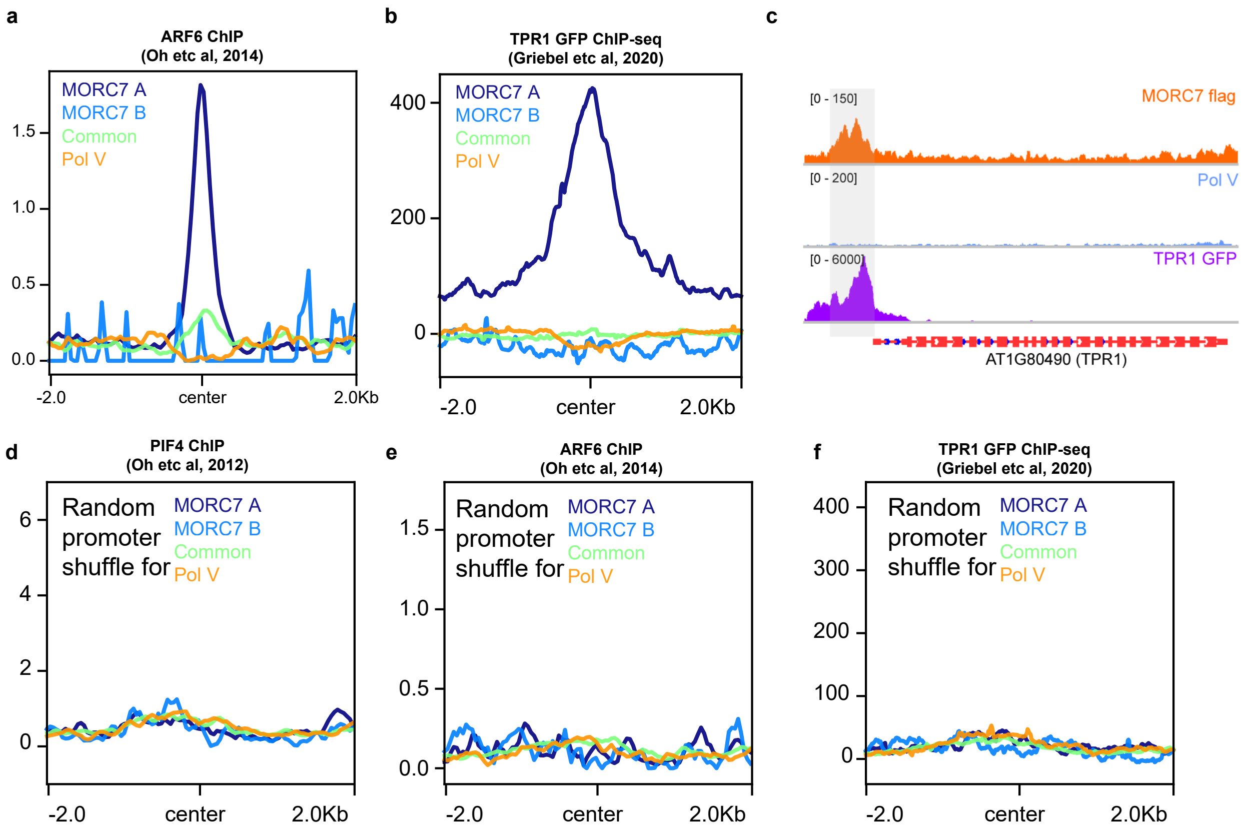

Fig. S4 MORC7 associates with some TFs. a. Metaplot of ARF6 ChIP-seq data [20] over MORC7A-unique, MORC7B-unique, MORC7-Pol V Common, and Pol V-unique regions. **b.** Metaplot of TPR1 ChIP-seq data [21] over MORC7A-unique, MORC7B-unique, MORC7-Pol V Common, and Pol V-unique regions. **c.** A screenshot showing MORC7A co-localization with TPR1. **d.** Metaplot of PIF4 ChIP-seq data over MORC7A-unique, MORC7B-unique, MORC7-Pol V Common, and Pol V-unique random promoter shuffle regions. **e.** Metaplot of ARF6 ChIP-seq data over MORC7A-unique, MORC7B-unique, MORC7-Pol V Common, and Pol V-unique random promoter shuffle regions. **f.** Metaplot of TPR1 ChIP-seq data over MORC7A-unique, MORC7B-unique, MORC7-Pol V Common, and Pol V-unique random promoter shuffle regions.

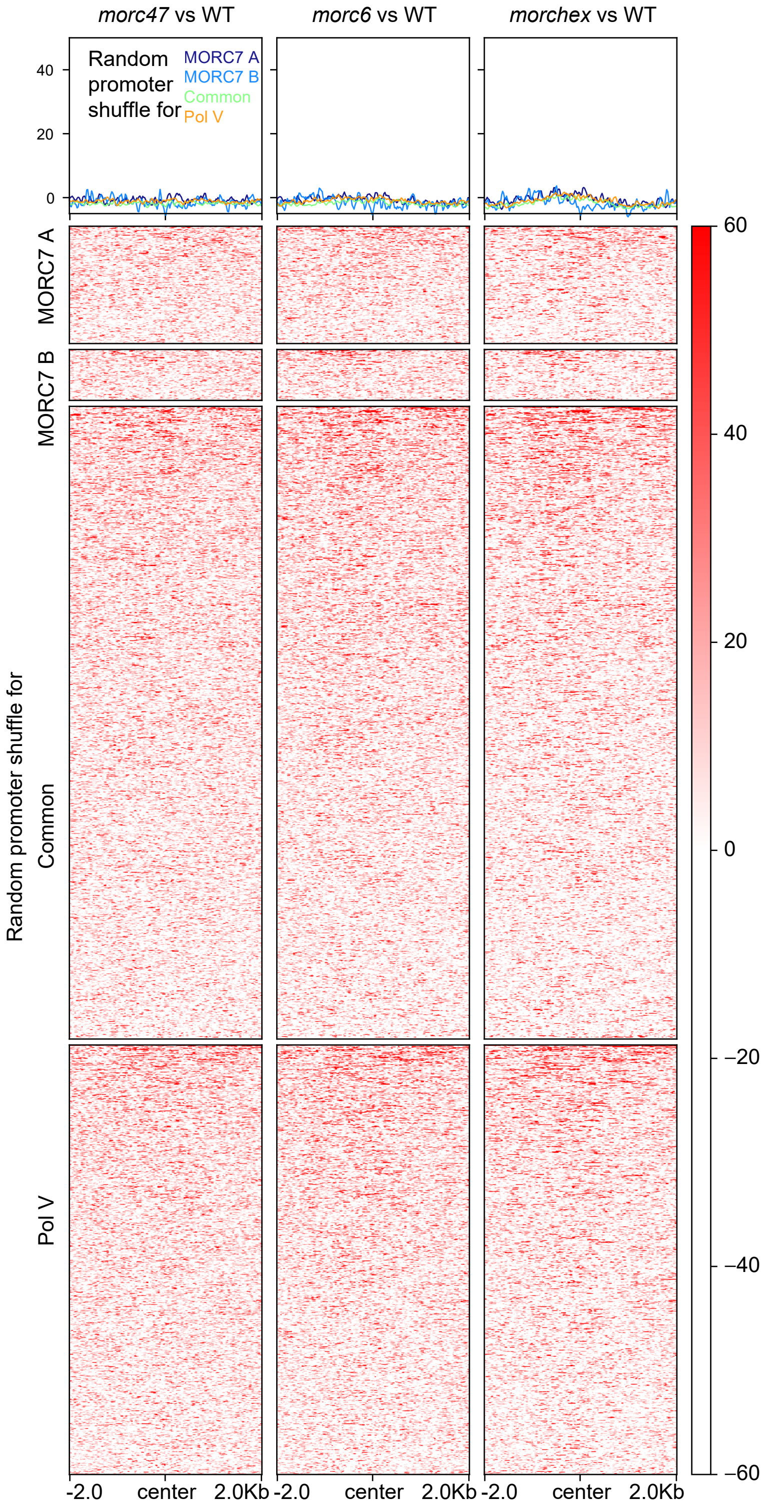

Fig. S5 Metaplot and heatmap showing chromatin accessibility changes in MORC7A-unique, MORC7B-unique, MORC7-Pol V Common, and Pol V-unique promoter random shuffle regions profiled by ATAC-seq.

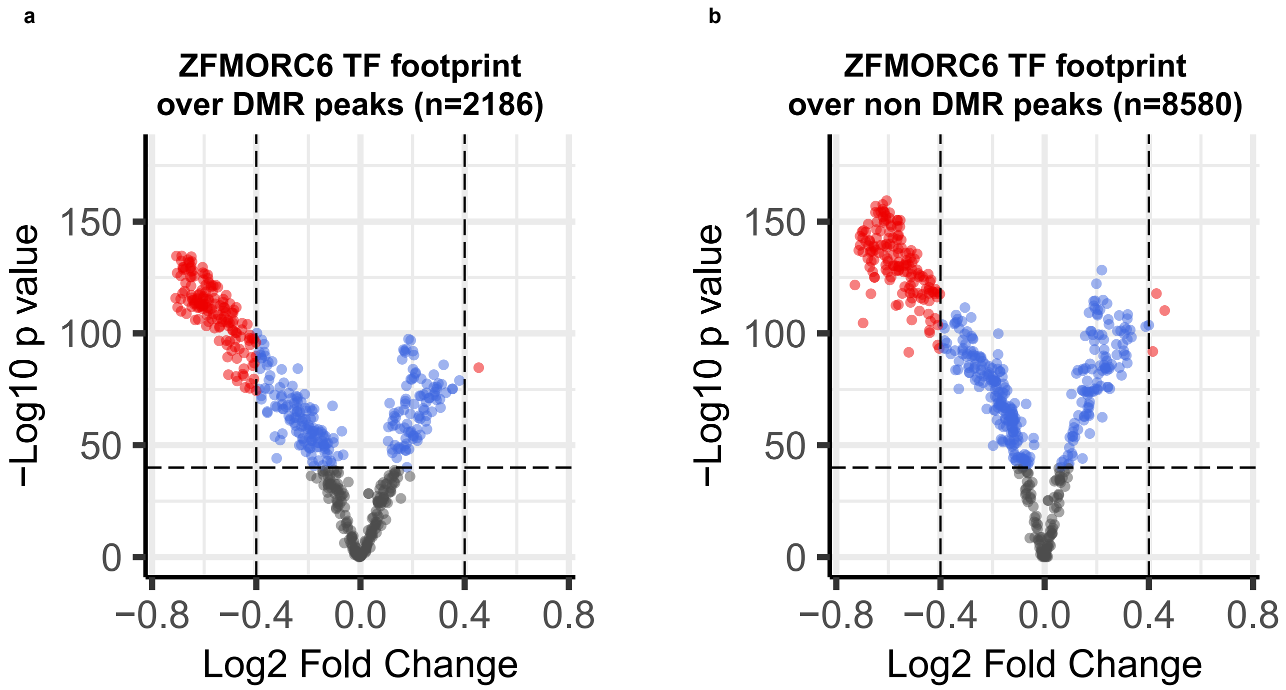

Fig. S6 a. Volcano plot showing TF changes for ZF off-target sites (n=2186) that gained DNA methylation comparing ZF-MORC6 and *fwa-4* plants. **b.** Volcano plot showing TF changes for ZF off-target sites (n=8560) that did not gain DNA methylation comparing ZF-MORC6 and *fwa-4* plants. P values were calculated by the two-sided Student's t-test.

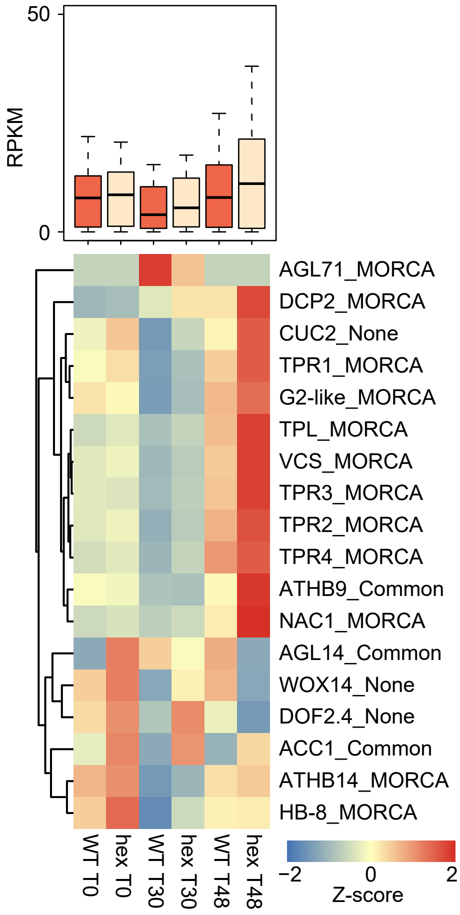

Fig. S7 Expression levels of genes in the primary shoot apical meristem specification pathway, with and without heat treatment, in Col-0 and *morchex* mutants.

TPL in WT

Motif	E-value	Site number
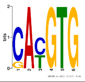	6.8e-119	1910
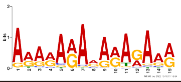	9.7e-092	4229
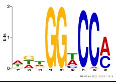	6.5e-066	1355
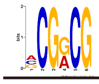	7.8e-031	1142
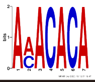	1.8e-001	664

TPL in *morchex*

Motif	E-value	Site number
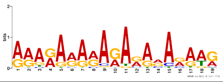	3.6e-128	3842
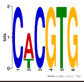	4.9e-108	1286
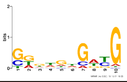	1.2e-015	11973
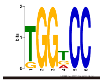	7.8e-024	1279
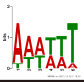	1.6e-010	9816

LUG in WT

Motif	E-value	Site number
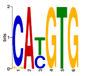	4.5e-050	702
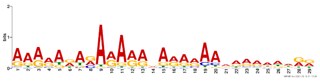	1.8e-025	1198
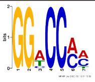	1.6e-018	534
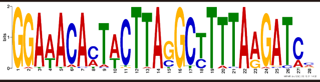	3.6e-004	33
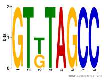	2.9e-003	49

LUG in *morchex*

Motif	E-value	Site number
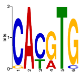	1.2e-095	1320
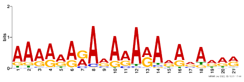	4.9e-069	2017
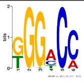	7.0e-056	1228
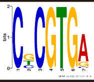	3.8e-019	282
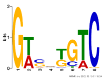	2.2e-005	1189

Fig. S8 Motif enrichment of TPL and LUG peaks identified in Col-0 or *morchex* mutant.

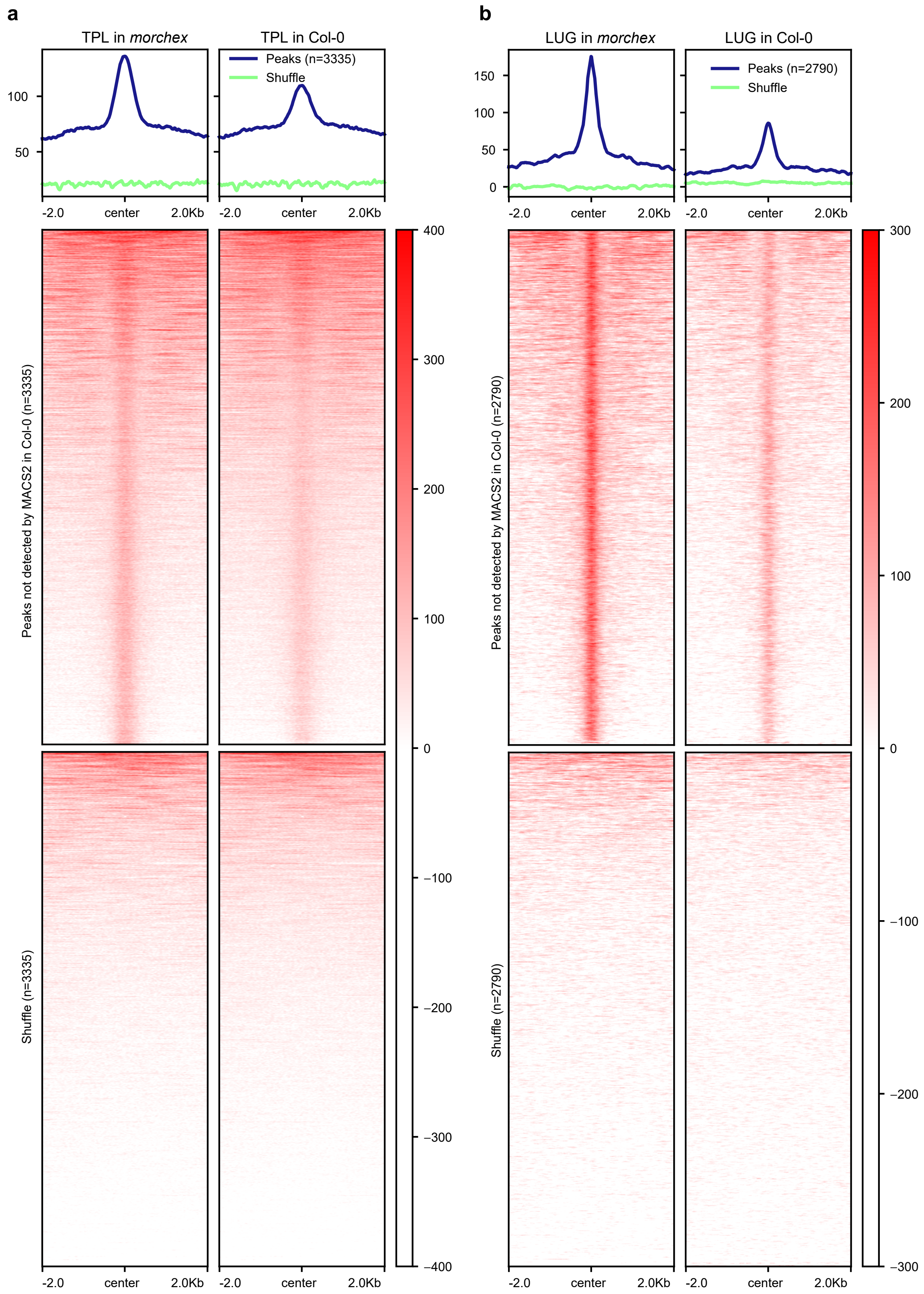

Fig. S9 a. Metaplot and heatmap showing TPL ChIP-seq signals over peaks detected in *morchex* mutant but not Col-0 by MACS2 (n=3335). **b.** Metaplot and heatmap showing LUG ChIP-seq signals over peaks detected in *morchex* mutant but not Col-0 by MACS2 (n=2790).
