# Supplementary material for: MORC proteins regulate transcription factor binding by mediating chromatin compaction in active chromatin regions
Source: Genome Biol. 2023 Apr 26;24:96. doi: 10.1186/s13059-023-02939-4 (PMC10131428; doi:10.1186/s13059-023-02939-4)
Supplement: Supplementary file 6 — Additional file 6: Table S5. Published ChIP-seq data used for ChromHMM states analysis. [file 13059_2023_2939_MOESM6_ESM.docx]

**Table S5** Published ChIP-seq data used for ChromHMM states analysis.

| **Data** | **SRA link** |
| --- | --- |
| Pol V | GSM2667837 [45] |
| H3K27me3 | SRR10905142 [47] |
| H3K27ac | SRR1509479 [50] |
| H3K9me2 | GSM2897853 [52] |
| H3K4me3 | SRR10905140 [48] |
| H3K9ac | GSM2366606 [54] |
| H4K16ac | GSM2882803 [56] |
| H3K4me1 | GSM3674621 [58] |
| H3K36me2 | GSM5684049 [60] |
| H3K36me3 | GSM5684053 [61] |
| Pol II | GSM5684057 [62] |
