# Supplementary material for: MORC proteins regulate transcription factor binding by mediating chromatin compaction in active chromatin regions
Source: Genome Biol. 2023 Apr 26;24:96. doi: 10.1186/s13059-023-02939-4 (PMC10131428; doi:10.1186/s13059-023-02939-4)
Supplement: Supplementary file 7 — Additional file 7. Review history. [file 13059_2023_2939_MOESM7_ESM.docx]

Review History

**First round of review**

**Reviewer 1**

**Were you able to assess all statistics in the manuscript, including the appropriateness of statistical tests used?**

Yes, and I have assessed the statistics in my report.

**Comments to author:**

The manuscript presented by Zhong and colleagues revealed RdDM-independent functions of MORC proteins in Arabidopsis. By performing the ChIP-seq experiment of MORC7, the authors previously reported that ~20% of MORC7 binding sites are devoid of RdDM, although most MORC7 binding regions (80%) are overlapped with RdDM. This study focuses on these RdDM-depleted regions and found that a subset of MORC7-unique regions is located within an active chromatin compartment between genes. Furthermore, they discovered that MORC7 preferentially binds to TF promoters. Re-analysis of published crosslinked MORC7 IP-MS data revealed that MORC7 might directly or indirectly interact with lots of TFs. They found that MORC proteins compact chromatin and reduce DNA accessibility to transcription factors (TFs), thereby repressing gene expression, especially under stress conditions. This study revealed novel functions of MORC proteins in Arabidopsis. Overall, this manuscript is well written; the figures are sufficiently annotated, and I find them easy to follow.

Comments:

1. Previous studies reported that SUVH2/9 can directly interact with MORC proteins and recruit MORC proteins to the RdDM loci and facilitate the efficiency of the RdDM pathway and gene silencing. How are MORC proteins recruited to the RdDM-independent regions? Can specific TFs, directly interacting with MORCs, recruit MORCs to the RdDM-independent regions?

2. Through re-analysis of the cross-linked IP-MS data and analysis of the ChIP-seq data, the authors claimed that MORC7 closely co-localizes with some TFs. Did the authors test whether these TFs can directly interact with MORC proteins? During analysis of the PIF4, ARF6, ad TRP1 ChIPseq data, there needs a random promoter control.

3. In Figure 3C, the authors tested MORC7 co-localization with 200 TFs. How many TFs are in the list of Table S2? In addition, since MORC7A is in promoter regions, it is expected that MORC7A co-localized with TF.

4. Figure 4A lacks a random gene promoter control.

5. Does the morc mutants show any phenotype under heat-stressed condition?

6. In Figures 5A and 5H, the difference between WT and mutant is significant enough?

**Reviewer 2**

**Were you able to assess all statistics in the manuscript, including the appropriateness of statistical tests used?**

Yes, and I have assessed the statistics in my report.

**Comments to author:**

Review of "MORC proteins regulate transcription factor binding by mediating chromatin compaction in active chromatin regions" by Zhong et al.

The evolutionarily conserved Microrchidia (MORC) ATPases are required for gene silencing and chromatin compaction in both plant and animal cells. In Arabidopsis, MORC proteins promote RNA-directed DNA methylation (RdDM) to establish gene silencing, yet it has been shown that they can also act downstream of DNA methylation. The molecular mechanisms of the RdDM-independent roles of MORC have yet to be elucidated. To investigate this, the authors focus on sites where MORC7 binds independently of RdDM (RdDM-depleted regions). They find that MORC7 forms peaks at promoters in active chromatin regions, as well as heterochromatic regions (MORC7A and MORC7B, respectively). MORC7A associates with transcription factors and seems to affect their binding by reducing chromatin accessibility in unmethylated promoter regions. By analyzing the TFs and expression data of nearby genes using the morchex mutant, the authors conclude that MORC proteins are likely to regulate expression of genes required for stress responses.

Critique: The work described in this manuscript is interesting and conclusions are supported by the results presented. The following suggestions should be addressed before acceptance of this work for publication in Genome Biology.

Specific comments:

1 The authors have compared histone modifications and DNA methylation levels over MORC7A-unique, MORC7B-unique, MORC7-Pol V Common, and Pol V-unique regions, and found that MORC7A-unique are enriched at active chromatin compartment independently with RdDM. I would suggest the authors compare small RNA levels in four groups and probably in some RdDM mutants to further support this conclusion.

2 Using gain of function ZF targeting assay, the authors demonstrated that targeting MORC protein to chromatin inhibits binding activity of TFs. While since targeting of MORC6 also triggers DNA methylation on some off-target sites according to previous study published by Gallego-Bartolome et al, 2019 Cell, ZFMORC6 TF footprint analysis should separate RdDM dependent and independent sites by comparing DNA methylation levels over these off-target sites.

3 The observation that the binding activity of TPL and LUG are increased over MORC7A-unique regions in morc mutant is very interesting, which directly supports the conclusions from ATAC-seq TF footprint analysis. Ideally further investigations could characterize the changes of TPL and LUG, such as binding motif enrichment and identification of new binding sites in other genomic regions.

**Authors Response**

**Point-by-point responses to the reviewers’ comments:**

*We thank the two reviewers for their positive comments and constructive suggestions. We have revised our manuscript according to the reviewers’ recommendations and provided our point-to-point response below* ***with referee comments in bold type*** *and our response in regular type.*

**Reviewer #1: The manuscript presented by Zhong and colleagues revealed RdDM-independent functions of MORC proteins in Arabidopsis. By performing the ChIP-seq experiment of MORC7, the authors previously reported that ~20% of MORC7 binding sites are devoid of RdDM, although most MORC7 binding regions (80%) are overlapped with RdDM. This study focuses on these RdDM-depleted regions and found that a subset of MORC7-unique regions is located within an active chromatin compartment between genes. Furthermore, they discovered that MORC7 preferentially binds to TF promoters. Re-analysis of published crosslinked MORC7 IP-MS data revealed that MORC7 might directly or indirectly interact with lots of TFs. They found that MORC proteins compact chromatin and reduce DNA accessibility to transcription factors (TFs), thereby repressing gene expression, especially under stress conditions. This study revealed novel functions of MORC proteins in Arabidopsis. Overall, this manuscript is well written; the figures are sufficiently annotated, and I find them easy to follow.**

*Reply: Thank you for these positive comments!*

**Comments:
1. Previous studies reported that SUVH2/9 can directly interact with MORC proteins and recruit MORC proteins to the RdDM loci and facilitate the efficiency of the RdDM pathway and gene silencing. How are MORC proteins recruited to the RdDM-independent regions? Can specific TFs, directly interacting with MORCs, recruit MORCs to the RdDM-independent regions?**

*Reply: Thank you for this question. Our current data cannot tell us exactly how MORC proteins are recruited to these RdDM-independent regions. We have added discussion on this point in the Discussion section as “A previous study suggested that the proteins SUVH2 and SUVH9 bind to methylated DNA and recruit MORC proteins to RdDM loci to facilitate the efficiency of RdDM and gene silencing [26]. However, the question of how MORC proteins are recruited regions devoid of RdDM remains to be answered”.*

**2. Through re-analysis of the cross-linked IP-MS data and analysis of the ChIP-seq data, the authors claimed that MORC7 closely co-localizes with some TFs. Did the authors test whether these TFs can directly interact with MORC proteins? During analysis of the PIF4, ARF6, ad TRP1 ChIPseq data, there needs a random promoter control.**

*Reply: Since the interaction data is from IP-MS experiments, we do not know which of the interactions might be direct and which might indirect.* *We did test the interaction between MORC7 and SEU and LUG by transient expression in tobacco followed by Co-IP. However, we did not observe an interaction between MORC and these TFs in this system. This suggests that these interactions are either indirect, or are weak or transient. Because this data was negative, we have not added it to the manuscript.*

*Thank you for this excellent suggestion and we have now added a random promoter control for PIF4, ARF6, and TRP1 ChIP-seq data. As shown below and in revised Supplementary 4, we did not detect significant enrichment of PIF4, ARF6, and TRP1 over random promoter controls (d-f).*

**Supplementary Fig. 4 MORC7 associates with some TFs. d.** Metaplot of PIF4 ChIP-seq data over MORC7A-unique, MORC7B-unique, MORC7-Pol V Common, and Pol V-unique random promoter shuffle regions. **e.** Metaplot of ARF6 ChIP-seq data over MORC7A-unique, MORC7B-unique, MORC7-Pol V Common, and Pol V-unique random promoter shuffle regions. **f.** Metaplot of TPR1 ChIP-seq data over MORC7A-unique, MORC7B-unique, MORC7-Pol V Common, and Pol V-unique random promoter shuffle regions.

**3. In Figure 3C, the authors tested MORC7 co-localization with 200 TFs. How many TFs are in the list of Table S2? In addition, since MORC7A is in promoter regions, it is expected that MORC7A co-localized with TF.**

*Reply: For the TFs characterized with DAP-seq in Figure 3C, 23 out of 68 TFs were identified in the MORC7 crosslinked IP-MS data (Supplementary Table 2). This information is now available in the last column of Supplementary Table 2.*

*For the co-localization analysis, we certainly agree that since MORC7A peaks are in promoters, and transcription factor binding sites are in promoters, this will automatically give a correlation between MORC7A peaks and transcription factor binding sites. What we think is more significant, as shown in Figure 3C is that MORC7A peaks are co-localized with particular transcription factor binding sites, but not with others. We have tried to clarify this in the text as “We found that MORC7A showed stronger co-localization with TFs compared to MORC7B, MORC7-Pol V common and Pol V-unique regions, and also shows strong co-localization with some TF binding sites but not others (Fig. 3c)”.*

**4. Figure 4A lacks a random gene promoter control.**

*Reply: Thank you for this suggestion. We produced a set of random control gene promoters through random shuffling of -1000~0bp promoter regions. We used these regions for the same analysis as Figure 4A. As shown below, the random regions did not show significant changes of chromatin accessibilities. This result has been added into Supplementary Figure 5.*

**Supplementary Fig. 5** Metaplot showing chromatin accessibility changes in MORC7A-unique, MORC7B-unique, MORC7-Pol V Common, and Pol V-unique promoter random shuffle regions profiled by ATAC-seq.

**5. Does the morc mutants show any phenotype under heat-stressed condition?**
*Reply: We applied 2 hours 37°C heat treatment on wild type and morchex mutant. We did not observe obvious phenotypic differences between wildtype and mutant, perhaps due to the short duration of treatment. Because these results were negative, these were not included in the paper. In this study, we mainly focused on gene expression changes between wild-type and mutant. As shown in Figure 4c and 4, we detected more upregulated genes and TEs after heat treatment in morchex.*

**6. In Figures 5A and 5H, the difference between WT and mutant is significant enough?**

*Reply: Thank you for pointing this out.* *We added statistical analysis between WT and morchex in Figure 5A and H. For Figure 5A, the p values appear to be significant. For Figure 5H, the different is significant for the heat treatment.*

**Reviewer #2: Review of "MORC proteins regulate transcription factor binding by mediating chromatin compaction in active chromatin regions" by Zhong et al.

The evolutionarily conserved Microrchidia (MORC) ATPases are required for gene silencing and chromatin compaction in both plant and animal cells. In Arabidopsis, MORC proteins promote RNA-directed DNA methylation (RdDM) to establish gene silencing, yet it has been shown that they can also act downstream of DNA methylation. The molecular mechanisms of the RdDM-independent roles of MORC have yet to be elucidated. To investigate this, the authors focus on sites where MORC7 binds independently of RdDM (RdDM-depleted regions). They find that MORC7 forms peaks at promoters in active chromatin regions, as well as heterochromatic regions (MORC7A and MORC7B, respectively). MORC7A associates with transcription factors and seems to affect their binding by reducing chromatin accessibility in unmethylated promoter regions. By analyzing the TFs and expression data of nearby genes using the morchex mutant, the authors conclude that MORC proteins are likely to regulate expression of genes required for stress responses.**

**Critique: The work described in this manuscript is interesting and conclusions are supported by the results presented.  The following suggestions should be addressed before acceptance of this work for publication in Genome Biology.**

*Reply: Thank you for these positive comments!*

**Specific comments:
1 The authors have compared histone modifications and DNA methylation levels over MORC7A-unique, MORC7B-unique, MORC7-Pol V Common, and Pol V-unique regions, and found that MORC7A-unique are enriched at active chromatin compartment independently with RdDM. I would suggest the authors compare small RNA levels in four groups and probably in some RdDM mutants to further support this conclusion.**

*Reply: Thank you for this suggestion. We analyzed 21nt, 22nt, and 24nt small RNA levels in four groups of peaks and analyzed their levels in mutants of the RdDM pathway. As shown below and in Supplementary Figure 2, we found that the level of 21nt, 22nt, and 24nt small RNAs over MORC7A group are extremely low, consistent with low DNA methylation levels at this region. In addition, we analyzed small RNA data of dcl234, nrpd1, nrpd1e1, and rdr2 mutants, and found that small RNA levels are dramatically reduced at these regions, particularly at Common and Pol V unique sites, which further supports our conclusion that MORCA is enriched at active chromatin regions.*

**Supplementary Fig. 2 Small RNA data over MORC7 peaks.** Boxplot showing 21-nt, 22-nt, and 24-nt siRNA in Col-0, *dcl234*, *nrpd1*, *nrpd1 nrpe1*, and *rdr2* mutants [31] over MORC7A-unique, MORC7B-unique, MORC7-Pol V Common, and Pol V-unique regions.

**2 Using gain of function ZF targeting assay, the authors demonstrated that targeting MORC protein to chromatin inhibits binding activity of TFs. While since targeting of MORC6 also triggers DNA methylation on some off-target sites according to previous study published by Gallego-Bartolome et al, 2019 Cell, ZFMORC6 TF footprint analysis should separate RdDM dependent and independent sites by comparing DNA methylation levels over these off-target sites.**

*Reply: Thank you for this suggestion. We analyzed transcription factor footprint over ZF sites that gain DNA methylation (n=2186) and that do not gain DNA methylation sites (n=8580). We found that TF footprints are significantly downregulated in both group of sites, suggesting that the inhibition of TF footprints mediated by MORC proteins are DNA methylation independent. This has been provided as a new supplementary Figure A, also shown below.*

**Supplementary Fig. 6 a.** Volcano plot showing TF changes for ZF off-target sites (n=2186) that gained DNA methylation comparing ZF-MORC6 and *fwa-4* plants. **b.** Volcano plot showing TF changes for ZF off-target sites (n=8560) that did not gain DNA methylation comparing ZF-MORC6 and *fwa-4* plants. P values were calculated by the two-sided Student's t-test.

**3 The observation that the binding activity of TPL and LUG are increased over MORC7A-unique regions in morc mutant is very interesting, which directly supports the conclusions from ATAC-seq TF footprint analysis. Ideally further investigations could characterize the changes of TPL and LUG, such as binding motif enrichment and identification of new binding sites in other genomic regions.**

*Reply: Thanks for this suggestion. We looked at motif enrichment over TPL and LUG peaks identified in wild-type and morchex backgrounds from our ChiP-seq data. While most of the peaks have similar motif enrichment between wild type and morchex, found 3335 peaks specifically detected in morchex background by MACS2. Metaplot of TPL and LUG ChIP-seq data suggested that these peaks also have ChIP-seq signal in wild type with lower intensity, indicating that MORC proteins affect binding strength of TPL and LUG. These results are now in new Supplementary Figure 8 and 9.*

**Supplementary Fig. 8** Motif enrichment of TPL and LUG peaks identified in Col-0 or *morchex* mutant.

**Supplementary Fig. 9** **a.** Metaplot showing TPL ChIP-seq signals over peaks detected in *morchex* mutant but not Col-0 by MACS2 (n=3335). **b.** Metaplot showing LUG ChIP-seq signals over peaks detected in *morchex* mutant but not Col-0 by MACS2 (n=2790).

**Reviewer 1**

The authors have successfully addressed all my issues.

**Reviewer 2**

The authors have addressed all my comments and the manuscript is now ready for publication.

**Authors Response**

**Point-by-point responses to the reviewers’ comments:**

Reviewer #1: The authors have successfully addressed all my issues.

*Reply: We thank again the reviewer for reviewing our manuscript.*

Reviewer #2: The authors have addressed all my comments and the manuscript is now ready for publication

*Reply: We thank again the reviewer for reviewing our manuscript.*
